# Supplementary material for: Advancing the application of pXRF for animal samples
Source: PLoS One. 2024 Dec 5;19(12):e0297830. doi: 10.1371/journal.pone.0297830 (PMC11620409; doi:10.1371/journal.pone.0297830)
Supplement: Attachment — Submitted filename: Methods paper_reviewers comments.docx [file pone.0297830.s005.docx]

Dawid Surmik
Academic Editor
PLOS ONE

3^rd^ June 2024

Re: Re-submission of PONE-D-24-01800 Advancing the application of pXRF for animal samples.

Dear Dr Surmik,

Please find following our responses to the reviewer comments on our manuscript. We are very grateful to our reviewers for their comments as they have significantly improved our manuscript and our understanding of this highly complex topic. As you will see we have taken every effort to meet the reviewers requests and detailed our responses below and we hope our revised manuscript is now suitable for publication.

Kind regards,

Kate Brandis

Reviewers' comments:

Reviewer's Responses to Questions

**Comments to the Author**

1. Is the manuscript technically sound, and do the data support the conclusions?

The manuscript must describe a technically sound piece of scientific research with data that supports the conclusions. Experiments must have been conducted rigorously, with appropriate controls, replication, and sample sizes. The conclusions must be drawn appropriately based on the data presented.

Reviewer #1: Yes

Reviewer #2: No

Please see our detailed responses to reviewers’ comments below, we feel we have addressed reviewers 2’s valid concerns and the manuscript now meets this criterion.

2. Has the statistical analysis been performed appropriately and rigorously?

Reviewer #1: Yes

Reviewer #2: No

Please see our detailed responses to reviewers’ comments below, we feel we have addressed reviewers 2’s valid concerns and the manuscript now meets this criterion.

 3. Have the authors made all data underlying the findings in their manuscript fully available?

The PLOS Data policy requires authors to make all data underlying the findings described in their manuscript fully available without restriction, with rare exception (please refer to the Data Availability Statement in the manuscript PDF file). The data should be provided as part of the manuscript or its supporting information, or deposited to a public repository. For example, in addition to summary statistics, the data points behind means, medians and variance measures should be available. If there are restrictions on publicly sharing data—e.g. participant privacy or use of data from a third party—those must be specified.

Reviewer #1: Yes

Reviewer #2: Yes

4. Is the manuscript presented in an intelligible fashion and written in standard English?

PLOS ONE does not copyedit accepted manuscripts, so the language in submitted articles must be clear, correct, and unambiguous. Any typographical or grammatical errors should be corrected at revision, so please note any specific errors here.

Reviewer #1: Yes

Reviewer #2: Yes

5. Review Comments to the Author

Please use the space provided to explain your answers to the questions above. You may also include additional comments for the author, including concerns about dual publication, research ethics, or publication ethics. (Please upload your review as an attachment if it exceeds 20,000 characters)

**Reviewer #1:** The article titled "ADVANCING THE APPLICATION OF pXRF FOR BIOLOGICAL SAMPLES" is undeniably an important work that addresses the application of portable X-ray fluorescence (pXRF) technology for analyzing biological samples, focusing on animals. It is clear from the review that this study is necessary and holds substantial value for publication due to its contributions to the field. However, several areas require attention and improvement to enhance the clarity, accuracy, and overall impact of the work.

Firstly, the title suggests a broader review (biology samples) rather than an experimental study limited to animal tissues. It would be beneficial to refine the title to accurately reflect the scope of the samples analyzed or to adjust the content to match the broad implication of the current title. This would ensure readers have a clear expectation of the article's content and scope. Animal Samples.

We have changed the title of the manuscript as suggested. It is now “ADVANCING THE APPLICATION OF pXRF FOR ANIMAL SAMPLES

The introduction lacks a comprehensive overview of the current literature on the application of pXRF in both biological and other types of samples, missing an opportunity to place the study within the context of existing research. Citing key studies, such mentioned below, would provide a solid background for readers:

https://doi.org/10.1016/j.trac.2023.117355

https://doi.org/10.1016/j.trac.2023.117165

https://doi.org/10.1016/j.jhazmat.2023.132167

As per the reviewers comments we have expanded the introduction from lines 58 – 64 to include additional key references and latest publications in this area. We have included the references noted by the reviewer and some additional, and recent studies that help to demonstrate range of applications of pXRF in both biological and other non-biological fields.

The methodological details appear to need more depth regarding the pXRF technique's application.
Please remember that portable X-ray fluorescence is not just a point and shoot method (see great review: https://doi.org/10.1016/j.envint.2019.105250)

We concur with the reviewer, the pXRF is not just a ‘point and shoot’ instrument, hence our manuscript identifying the type of issues that need to be considered when sampling animal tissues. We feel that our methods as described are adequate and repeatable. We have added some text to the methods section (line 146 onwards) regarding the analysis of diagnostic peaks and the determination of infinite thickness based on Reviewer 2’s comments.

Questions arise from the description provided, such as:
- the standards used for calibration, The Olympus Vanta is a self calibrating device. Calibration checks where done regularly throughout the testing process. The following text has been added at Line 126 “The instrument was factory calibrated by Olympus and calibration checks were performed throughout the sampling process using the onboard calibration option.”

- measurement modes, - Line 124 states the GeoChem3 mode was used.
- duration of measurements, Line 132 states the duration of measurements
- how the data, including fluorescence spectra and detection limits (LOD, LOQ), were handled? Any samples returning a measure below the LOD were removed from analyses. This has been noted in the text at Line 151.

Supplementary materials are not sufficient, readers want to know step by step methodology workflow and all related data (full excel with level, time duration, mode of measurement and etc).

We understand the reviewers’ comments, however we would argue that the step by step methodology is presented in the text. We had added some additional methods on data handling at lines 147-153.

All related data extends to over 495,000 data points for concentration data and 12,500,000 data points for the raw beam spectra data which we have noted is available on request from the authors, but we don’t feel if useful as a supplementary data set. We have provided key data in the supplementary that supports our results.

Addressing these questions would significantly strengthen the credibility and reproducibility of the study.

Additionally, the presentation of Latin names with spell-check underlines visible in Figure 1 suggests a need for a more professional approach to figure preparation. Utilizing dedicated graphic design software could enhance the visual quality of the publication.

These errors have been corrected and visual quality improved. All figures have been recreated using Adobe Illustrator updated and replaced.

While this study is fundamentally significant and intriguing, a major revision is required to address these concerns thoroughly. Enhancing the title's specificity, expanding the literature review, detailing the methodology more comprehensively, and improving figure quality would greatly benefit the manuscript's contribution to the field and its suitability for publication.

We thank the reviewer for their time and comments, we hope we have adequately addressed them.

**Reviewer #2:** The paper describes an analysis of different alternatives for the application of pXRF in biological tissues, aiming to show the simplicity of the technique and the wide range of potential applications. This point has been well established in the work, clearly showing that there are uses of pXRF in biology that have not yet been explored. Effectively, pXRF is very simple to use, but without careful analysis of the measurements, it is limited to a qualitative, usually preliminary study of more detailed laboratory studies. This work has some weaknesses, specifically in the data analysis.

On the one hand, the method used to identify infinite thickness samples needs to be improved as it is not sufficiently accurate. Effectively, if the silicon signal is successfully detected, then the sample is thin for silicon and for elements with atomic weight greater than silicon, as X-ray attenuation in the sample decreases as Z increases. However, if the silicon signal is not detected, it is difficult to conclude that the sample is infinite for two reasons: the XRF spectrometer's detection limit is low, and X-ray attenuation in the sample decreases as Z increases. In the first case, a high silicon detection limit leads to the conclusion that the sample is infinite when it is not actually the case. In the second case, because X-ray attenuation decreases drastically with Z, a thickness that may be considered infinite for silicon may not be so for heavier elements like iron, for example.

In summary, the methodology proposed by the authors effectively identifies samples of very thin thickness but does not allow for the precise identification of samples of infinite thickness. Therefore, it can erroneously endorse the elemental quantification of non-infinite thickness samples with the model of infinite thickness, leading to an underestimation of elemental concentrations. This error is serious because it may conclude that there are variations in the sample's composition when in reality, it's just variations in its thickness. One way to obtain more information about the sample thickness is to analyze the dispersion of the characteristic lines of the X-ray tube, in this case, the W L lines (See for example A. A. Markowicz and R. E. Van Grieken, Chapter 8. Quantification in XRF Analysis of Intermediate-Thickness Samples, in Handbook of x-ray spectrometry, Marcel Dekker, New York, 2002, 2nd edn.)

In other hand, when there are many overlapped XRF lines, many commercial software infer the possible presence of elements based on fractions of theoretical lines, which must be carefully reviewed. In this work has been reported elements that are rarely found in biological tissues, such as U, Th, Pr, La, Pb, Sb, Sn, Bi. I recommend including some representative XRF spectra of the samples to allow observation of which elements are directly detected and only using these elements in subsequent statistical analysis.

We are very grateful for the reviewer bringing these issues to our attention, it has significantly improved our manuscript and we have addressed both issues raised. We took the reviewers advice and reanalysed our data. To reassess if infinite thickness was being accurately detected we inspected the beam spectra data to analyse the dispersion of the characteristic lines of the X-ray tube, in this case, the L line for tungsten (W). We have revised as results as required based on the new analyses. We have amended our methods to remove reference to using silica to determine infinite thickness and now use reviewer 2’s suggested method (lines 153-156). We have also amended our results as necessary.

With regards to Reviewer 2’s comment on non-biologically relevant elements. Again, we reanalysed our data and checked for diagnostic peaks in the beam spectra data using 80s scans for each species group. We excluded any elements that did not match their Kɑ1 and Kß2 peaks. This approach allowed us to remove all non-biologically relevant elements. We have amended our methods to describe this approach (lines 146-150). We have also amended our results as necessary.

6. PLOS authors have the option to publish the peer review history of their article (What does this mean?). If published, this will include your full peer review and any attached files.


If you choose “no”, your identity will remain anonymous but your review may still be made public.


**Do you want your identity to be public for this peer review?** For information about this choice, including consent withdrawal, please see our Privacy Policy.

Reviewer #1: No

Reviewer #2: No
